# Review of proposed different irradiation methods to inactivate food‐processing viruses and microorganisms

**DOI:** 10.1002/fsn3.2539

**Published:** 2021-08-22

**Authors:** Sharifeh Shahi, Reza Khorvash, Mohammad Goli, Seyed Mohsen Ranjbaran, Afsaneh Najarian, Abdorreza Mohammadi Nafchi

**Affiliations:** ^1^ Department of Biomedical Engineering Isfahan (Khorasgan) Branch Islamic Azad University Isfahan Iran; ^2^ Laser and Biophotonics in Biotechnologies Research Center Isfahan (Khorasgan) Branch Islamic Azad University Isfahan Iran; ^3^ School of Medicine Isfahan University of Medical Sciences Isfahan Iran; ^4^ Department of Food Science and Technology Isfahan (Khorasgan) Branch Islamic Azad University Isfahan Iran; ^5^ Department of Food Science University of Guelph Guelph ON Canada; ^6^ Food Technology Division School of Industrial Technology Universiti Sains Malaysia Penang Malaysia; ^7^ Department of Food Science and Technology Damghan Branch Islamic Azad University Damghan Iran

**Keywords:** food processing, ionizing radiation, nonionizing radiation, viruses radiation‐inactivation, visible‐light radiation

## Abstract

Coronaviruses, which have been enveloped nonsegmented positive‐sense RNA viruses, were first mentioned in the mid‐1960s and can attack people as well as a wide range of animals (including mammals and birds). Three zoonotic coronaviruses have been identified as the cause of large‐scale epidemics over the last two decades: Middle East respiratory syndrome (MERS), severe acute respiratory syndrome (SARS), and swine acute diarrhea syndrome (SADS). Epithelial cells in the respiratory and gastrointestinal tract are the principal targeted cells, and viral shedding occurs via these systems in diverse ways such as through fomites, air, or feces. Patients infected with the novel coronavirus (2019‐nCoV) reported having visited the Wuhan seafood wholesale market shortly before disease onset. The clinical data on established 2019‐nCoV cases reported so far indicate a milder disease course than that described for patients with SARS‐CoV or MERS‐CoV. This study aimed to review radiation inactivation of these viruses in the food industry in three sections: visible light, ionizing radiation (alpha ray, beta ray, X‐ray, gamma ray, neutron, plasma, and ozone), and nonionizing radiation (microwave, ultraviolet, infrared, laser light, and radiofrequency). Due to the obvious possibility of human‐to‐human and animal‐to‐human transmission, using at least one of these three methods in food processing and packaging during coronavirus outbreaks will be critical for preventing further outbreaks at the community level.

## Graphical abstract

This study aimed to review radiation‐inactivation of these viruses in the food industry in three sections: visible light, ionizing radiation (alpha‐, beta‐, x‐, gamma‐ray, neutron, plasma, and ozone) and non‐ionizing radiation (microwave, ultra‐violet, infra‐red, laser light, and radiofrequency). Due to the obvious possibility of human‐to‐human and animal‐to‐human transmission, using at least one of these three methods in food processing and packaging during coronavirus outbreaks will be critical for preventing further outbreaks at the community level.

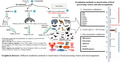

## 1 INTRODUCTION

Viruses are intracellular parasitic obligates. They do not have the biochemical machinery (organelles and enzymes) to survive outside of living cells. They have a simple structure, with a nucleocapsid made up of a genome (i.e., DNA or RNA) and proteins. Viruses are divided into two types: enveloped and nonenveloped viruses. The nucleocapsid is enclosed by a lipid membrane in enveloped viruses, which is acquired during virus assembly from infected cells. This lipid envelope contains moieties that aid virus attachment and entry during subsequent infection cycles, such as glycoproteins (modified proteins). This lipid membrane is absent in nonenveloped viruses. The nucleocapsid center is instead surrounded by other structural proteins that have been meticulously arranged into a spore‐like structure (Zia & Goli, 2021; Goli, 2020a, 2020b).

**FIGURE 1:**
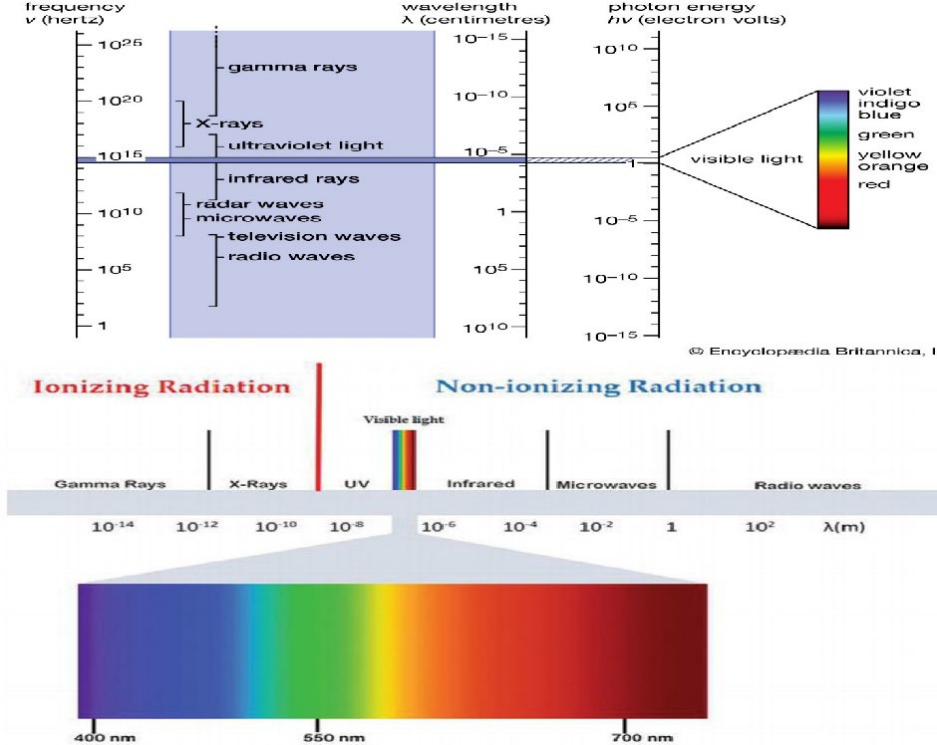
Electromagnetic spectrum (Lima et al., 2018)

To prevent microbial contamination and spoilage, food is packaged (Paidari & Ibrahim, 2021). Thermal processing is also an effective method for some sterilized food processing and inactivating foodborne viruses (Bosch et al., 2018). High‐pressure processing (HPP) compresses food suspended in liquid or gas, and releasing pressure indicates applicability for inactivating human‐CoV (Bosch et al., 2018). Treatment of packages, often made of natural or synthetic plastics, used in the aseptic processing of foods and pharmaceutical drugs, with gamma and electron radiation is now becoming a common practice, according to previous studies (Haji‐Saeid et al., 2007). Different portions of the electromagnetic radiation spectrum emit different types of radiant energy: radio waves, microwaves, infrared radiation, visible light, ultraviolet, X‐rays, and gamma rays. The wavelength, frequency, force of penetration, and other effects that these sources of energy have on biological systems (Lima et al., 2018). The regulated application of short‐wavelength radiation energy, such as gamma rays, accelerated electrons, and X‐rays, to food is known as radiation processing. Insect disinfestation of stored dried products; phytosanitation to resolve quarantine barriers in fruits and vegetables; inhibition of sprouting in tubers, bulbs, and rhizomes; delay in ripening and senescence of fruits; extension of shelf‐life by destruction of spoilage microbes; and elimination of pests and destruction of pathogens and parasites because of to the highly penetrating aspect of radiation energy are some of the goals that radiation processing of food may achieve (Lima et al., 2018). To ensure the microbiological, virological, and nutritional quality of food, several methods have been investigated by the food industry, including the use of different photochemical wavelength radiations, thermal processing, and pulsed electric fields. In photochemistry, the wavelength range is usually 100–1000 nm. When light photons with wavelengths longer than 1,000 nm are absorbed, they do not have enough energy to cause chemical change, while photons with wavelengths shorter than 100 nm have enough energy to cause ionization and molecular disturbances, which are typical of radiation chemistry. The visible light spectrum (Table 1), from 380 to 700 nm, includes some wavelengths, like blue, that have been emphasized for treatment or disinfection in food processing (Cheng et al., 2020). As shown in Figure 1, there are two types of radiation in the electromagnetic spectrum of wavelength range: ionizing (Table 2) and nonionizing radiation (Table 3) (Lima et al., 2018). Ionizing radiations are forms of energy emitted by atoms in the form of electromagnetic waves (such as gamma or X‐rays) or particles (such as neutrons, alpha, or beta).

The purpose of this review is to explain different irradiation methods for microorganism and virus inactivation in the food processing and packaging industry. Animal‐to‐human and subsequent human‐to‐human transmission is possible because animals, poultry, and their related products are potential carriers of various viruses. Different irradiation methods include visible light, ionizing radiation (such as alpha ray, beta ray, X‐ray, gamma ray, neutron, plasma, and ozone) and nonionizing radiation (microwave, ultraviolet (such as UV‐A, UV‐B, and UV‐C), infrared, and laser light). At least one of these three techniques, either alone or in combination with other preservation methods (such as thermal or nonthermal procedures), referred to in the food industry as Hurdle technology, would be critical in preventing greater coronavirus prevalence at the society level.

## 2 SPECTRUM OF VISIBLE LIGHT

The visible light spectrum is the portion of the electromagnetic spectrum that is visible to the naked eye, with wavelengths ranging from 380 to 700 nanometers (Figure 1, Table 1). Other sections of the spectrum have wavelengths that are either too large or too short and energetic for our biological limitations. The shortest wavelength is around 380 nanometers, while the longest wavelength is around 700 nanometers. The visible light spectrum also has many applications in biology, medicine, industry, and food processing. New research studies have found visible violet‐blue light with a wavelength in the area of 405 nm has significant germicidal effectiveness, and that the mode of action differs significantly from that of UV light due to the different wavelengths. UV light that is absorbed by the thymine and cytosine bases in viral RNA or bacterial DNA, resulting in cross‐linking and photoproducts that disrupt transcription and replication, contributing in mutations and death of cells (Carreres‐Prieto et al., 2019).

**TABLE 1:** Applications and observation of effects of visible light (λ = 380–700 nm) in the food industry

**Type**	**Application**	**Observation effects**	**References**
**Visible‐light**	Environmental decontamination Continuous disinfection of air and exposed surfaces in hospitals	Significant antimicrobial and antiviral properties against a wide range of bacterial, fungal, and viral pathogens by violet‐blue light (λ = 405 nm)	Felix et al., 2018
	Environmental decontamination technology and disinfection of the hospital environment with various combinations of photon fluxes and photoperiods of red, blue, and white light‐emitting diodes (LEDs) Cold storage of crops and foods in order to reduce thermal damage and degradation using LEDs in the visible‐light spectrum These sources preserve or enhance the nutritive quality of foods in the postharvest stage	Broad germicidal efficacy and destruction of a wide range of prokaryotic and eukaryotic microbial species, including resistant forms such as bacterial and fungal spores and pathogens	Mohidul et al., 2017; Souza et al., 2015

The antimicrobial effects of 405 nm light have applications in environmental decontamination, with a focus on hospital disinfection. A high‐intensity, narrow‐spectrum light environmental disinfection device was developed and tested in hospital isolation rooms for practical use. The results of the trials showed that this 405‐nm light system would disinfect the air and exposed surfaces in occupied areas of the hospital continuously, significantly improving normal cleaning and infection control procedures. Violet‐blue light, especially light with a wavelength of 405 nm, has potent antimicrobial and antiviral properties against a variety of bacterial, fungal, and viral pathogens. Though the germicidal effectiveness is less than that of UV light, this disadvantage is compensated for by its ability to be used safely and continuously in occupied spaces (Felix et al., 2018).

In the food industry, visible light emitting diodes (LEDs) have special properties that make them ideal for a variety of applications. Low radiant heat emissions; high monochromatic light emissions; electrical, luminous, and photon efficiency; long life expectancy; flexibility; and mechanical robustness are some of these properties. LEDs are ideal for cold storage applications because they minimize thermal damage and deterioration in crops and foods. Increased yields and nutritive quality of agricultural or horticultural products can be achieved by controlling the spectral composition of emitted light. LEDs have recently been used to conserve or improve the nutritional quality of foods during the postharvest stage, as well as to manipulate fruit ripening and minimize fungal infections. To inactivate pathogenic bacteria and viruses in food, LEDs can be combined with photosensitizers or photocatalysts. UV‐LEDs, which are quickly being produced, can also be used to successfully inactivate pathogens and preserve food during the postharvest stage. As a result, LEDs in the visible range (380–700 nm) minimize microbial contamination and provide a nonthermal or cold‐food‐safe solution that does not use chemical sanitizers or additives, accelerates bacterial tolerance, or alters postharvest ripening of vegetables and fruits. LEDs have the potential to be both cost‐effective and environmentally friendly. UV‐light LEDs are just as effective as UV‐C radiation at inactivating microorganisms, especially viruses (D’Souza et al., 2015; Mohidul et al., 2017).

## 3 IONIZING RADIATION

**FIGURE 2:**
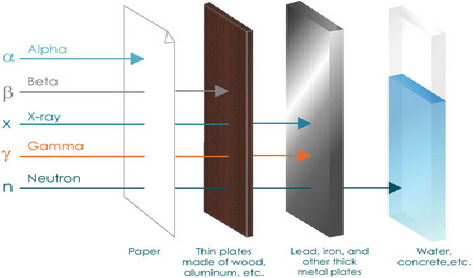
Type of ionizing radiations and penetration depth (Antonio‐Javier et al., 2018)

Ionizing radiations are widely used in the treatment of foods today. Ionizing radiation is so named because its frequency is high enough to displace electrons from atoms and molecules, converting them to electrical charges known as ions. High‐energy particles (such as alpha, protons, electrons, and neutrons) or electromagnetic waves (such as gamma rays and X‐rays) transmit ionizing radiation (Figure 2, Table 2). Only electron‐beam radiation and electromagnetic are used on food among the different types of ionizing radiation that exist. Radiation by emission of electrons is based on the transfer of energy by the acceleration of electrons, whereas electromagnetic radiation propagates in the form of waves. Irradiation of food is a physical treatment that involves exposing the product, which is already packed or in bulk, to regulated doses of ionizing radiation for sanitary, phytosanitary, and/or technical reasons. Radiation treatment and sterilization are especially common for fully packed, sealed items because they eliminate post‐sterilization packaging measures, reducing the risk of recontamination. The radiation compatibility of medical‐device parts, packaging materials, and foods must all be taken into account (Lima et al., 2018). Although irradiation is effective at preserving foods for the market, its efficacy against viruses is relative to the size of the virus, the suspension medium, the characteristics of the food product, and the exposure period. Most viruses are much more resistant to irradiation than vegetative bacteria, parasites, or fungi, possibly due to their smaller genome size and mostly single‐stranded RNA. Some studies have tested two main irradiation methods, gamma irradiation and electron beam (E‐beam), which both use high‐energy electrons. International regulatory agencies allow different doses depending on the type of food. However, the 4 kGy dose approved by the US FDA is unlikely to achieve viral reduction, and higher doses would be needed in most foods to achieve higher viral reductions. The stem‐cell‐derived human enteroid assay showed that gamma irradiating a human NoV GII.3 and GII.4 stool suspension for 8 kGy inactivates the viruses. When treated with an E‐beam, MNV tends to be more resistant than TuV in surrogate studies (Bosch et al., 2018). Traditional chemical or heat‐based sterilization methods have a number of disadvantages when compared to radiation‐based sterilization. However, others are not cost‐effective to use, and some can be harmful to one's health if used in food processing.

**TABLE 2:** Applications and observation of effects of ionization radiations in food processing and packaging

**Type**	**Applications**	**Observation effects**	**References**
**Alpha**	Not currently used for treatment of viruses or inactivation of microorganisms	Alpha particles interact strongly with matter.	Garcia‐Sanchez et al., 2018
**Beta**	High‐energy beta rays have a range of a few mm in tissue or matter and are increasingly being used for radio‐immune therapy of cancer Not economical in current food‐processing applications	Beta emitters have been successfully used for treatment of experimental fungal, bacterial, and viral infections, with transient or no hematologic toxicity	Garcia‐Sanchez et al., 2018; Kassis, 2008; Dadachova, 2008
**X‐ ray**	X‐rays used for sterilization can be more penetrating than either gamma‐rays or electron beams	Inactivates human viruses	Qin, 2016; Silvestre et al., 2017
**Gamma**	Surface and air treatment and cleaning; effective in inactivating microorganisms for food preservation and packaging materials	Inactivates human viruses such as NoV, GII.3, and GII.4	Lima et al., 2018; Silvestre et al., 2017; Al‐Ani and Al‐Khalidy, 2006
**E‐beam**	Because penetration ability of electrons is lower than that of gamma rays, e‐beam sterilization is limited to lower‐density or smaller products	Inactivates human viruses	Lima et al., 2018; Silvestre et al., 2017; Al‐Ani and Al‐Khalidy, 2006
**Neutron**	Inactivation of viral pathogens for sterilization Used in manufacturing of biological reagents, including production of noninfectious viral antigens	Frozen samples of influenza X31/H3N2 and PR8/H1N1 were exposed to gamma and neutron radiation and inactivated	Garcia‐Sanchez et al., 2018; Lowy et al., 2001
**Plasma**	Diseases such as HIV, the common cold, and even cancer	Nonthermal plasma treatment induces different effects in surface decontamination of foodstuffs, deposition, induced polymerization, grafting, cleaning, functionalization, or crosslinking in the emerging field of antimicrobial, bioactive food‐packaging materials	Morozov, 2013; Silvestre et al., 2017; Bartos et al., 2017; Pankaj et al., 2014; Filipic et al., 2020
**Ozone**	Ozone gas reacts swiftly and effectively on all strains of viruses, showing faster activity than treatments that use chlorine or hydrogen peroxide Inactivation of human NoV, HAV, TuV, and MNV proportional to processing temperatures and high‐pressure processing	This alternative treatment is based on compressing food suspended in liquid or gas and releasing high pressure quickly. Strong capacity for disinfection and sterilization in the food processing, hospitality, and other industries	Cheng et al., 2020; Augusto Goncalves, 2009; Quevedo et al., 2020

### 3.1 Alpha radiation

When an atom undergoes nuclear decay, it emits an alpha particle, which is made up of two protons and two neutrons (basically the nucleus of a helium‐4 atom), transforming the originating atom into one with an atomic number 2 less and atomic weight 4 less than before. Alpha particles interact intensely with matter due to their charge and mass, and only fly a few centimeters in the air. Polonium‐210, radon‐222, radium‐226, thorium‐232, plutonium‐236, and uranium‐238 are examples of alpha emitters. Alpha particles are unable to reach the outer layer of dead human skin cells, but they are capable of causing significant cell damage if an alpha‐emitting material is consumed in food or air. As a result, in the food industry, this form of radiation is rarely used for packaging and disinfection (Garcia‐Sanchez et al., 2018).

### 3.2 Beta radiation

Various high‐energy isotopes, such as strontium‐90, cesium‐173, iridium‐192, gold‐198, and radon‐222 emit β‐radiation, which is a high‐speed, high‐energy electron or positron. Positive and negative beta particles interact with the outer shells of the atoms they move through, exciting and ionizing them as they migrate through a substance. Ion pairs are strewn around the beta particle's path. It reaches the end of its range when it has expended all of its initial energy. The greater the beta particle's initial energy, the greater its range. The density of the substance through which it passes is inversely proportional to this range. In tissue, the most energetic beta rays have a range of just a few millimeters (Kassis, 2008). Beta emitters, such as 90Y, are now widely used and recorded for cancer care (Jodal, 2009). Bismuth‐213, a beta‐emitter (97%) and an alpha‐emitter (3%) with a physical half‐life of 46 min, decays to the short‐lived alpha‐emitter polonium‐213. It has been widely used for treating experimental viral, bacterial, and fungal infections with intermittent or no hematologic toxicity, and it has been successfully used for radio‐immunotherapy (RIT) of cancer over the last decade in both clinical and pre‐clinical work (Dadachova, 2008).

### 3.3 X‐ray radiation

X‐rays are produced by electron beam accelerators for use in sterilization. When high‐energy electrons from the accelerator collide with high‐atomic‐number nuclei, such as tantalum or tungsten atoms, X‐rays are emitted. Bremsstrahlung process occurs when an electron decelerates when it passes through the nucleus, resulting in the emission of X‐rays. Commercially, electron energies of 5–7 MeV are used; the energies of the resulting X‐rays range from zero to the energy of the electron beam (Qin, 2016). In practice, X‐rays are more penetrating than gamma rays or electron beams for sterilization. They are primarily aimed at sterilization. As a result, a coordinated stream of X‐rays is directed at the target product, and several rows of items can be sterilized at the same time. X‐ray sterilization has the highest dose uniformity level of all radiation sterilization techniques (minimum and maximum dose ratio required to optimize for irradiation‐sensitive materials for minimizing degradation). As a result, electron‐beam irradiation, which is produced by electron‐beam accelerators and capable of causing biological harm, can be used to sterilize (Qin, 2016). Sample penetration depth, exposure time needed for successful sterilization, and product compatibility vary between gamma and e‐beam irradiation. Since electrons have a lower penetration potential than gamma rays, E‐beam sterilization can only be used on lower‐density or smaller items. As opposed to gamma radiation sterilization, X‐ray sterilization may use higher doses and shorter treatment periods (seconds versus. min/hours), allowing for higher efficiency and throughput and minimizing harmful effects on treated items (Silvestre et al., 2017). E‐beam sterilization is comparable to or less costly than gamma sterilization in terms of cost.

### 3.4 Gamma radiation

Gamma irradiation has many benefits, including nonpolluting application, efficacy at room temperature, and some process control versatility. Since the photon energy is strong enough to pass into materials, gamma irradiation is not a surface treatment. Only the surface of a polymeric packaging material can be modified under such experimental conditions (Ahari et al., 2016; Sohrabi Haghdoust et al., 2019). Following gamma irradiation, functional groups may bind to a surface, enabling the material to immobilize enzymes or other bioactive organisms (Silvestre et al., 2017). Gamma rays have a frequency of above 1,019 Hz, implying wavelengths of less than 10 × 10^–12^ m. Radiation equipment consists of a single high‐energy radiation source (e.g., isotope source) that emits gamma rays, or, less commonly, equipment that emits high electron‐beam energy. The excited nuclei of radioactive elements such as cesium‐137 and cobalt‐60 emit gamma radiation (Lima et al., 2018). Inactivating microorganisms with gamma radiation is very successful. Since each product's bacterial count should be as low as possible, products should be treated as little as possible during the manufacturing process. The facility should be clean and dry, ventilated with fresh air, and constructed and furnished in a way that allows for thorough cleaning on a regular basis. The morphology and properties of packaging materials are also affected by electron‐beam or gamma irradiation used for food preservation (Lima et al., 2018). At doses more than 10 kGy (virus clearance between 10 and 100 kGy), gamma radiation may have a fatal effect on COVID RNA decrease in the virus mono‐chain. It is possible that when the radiation dosage rises, the COVID particles' proteins, as well as the quantity of their genes, deteriorate, resulting in fewer infections (Sanglier Contreras et al., 2020). However, gamma radiation sterilization has many benefits, including low heat generation, irradiation of packaged or frozen foods, and improvements in food nutritional value that are comparable to or inferior to other preservation methods. The high initial cost of gamma radiation, as well as the difficulty in establishing the correct doses, since some doses will kill certain insects while others are still alive, are disadvantages. Despite the advantages, a number of obstacles remain, preventing irradiated foods from being widely available. These barriers are primarily linked to the cost of their usage and consumer opposition due to a lack of knowledge on food‐induced radioactivity. Crosslinking occurs in situ and at lower temperatures in the solid state of finished materials, eliminating the need for heating or melting of polymers, which is an advantage of electron and gamma irradiation over chemical processing of polymers. Ionizing radiation, such as low‐energy (less 25 keV) electron beams, can be used in materials manufacturing and processing to enhance adhesion properties by treating the surface (Silvestre et al., 2017). According to another study, gamma radiation is the most commonly used form of radiation in wastewater treatment. The exposure period for a given dose is determined by the strength of a gamma‐ray source. The bulk density and irradiator configuration used in wastewater treatment are affected by certain factors such as the water geometry and the presence of solids in the water (Al‐Ani et al., 2006).

### 3.5 Neutron radiation

Neutron radiation is made up of a free neutron due to spontaneous or induced nuclear fission, can fly hundreds or even thousands of meters in the air. When a hydrogen‐rich substance, such as concrete or water, blocks them, they are essentially prevented. Since neutrons lack the ability to actively ionize an atom due to their lack of charge, they are most generally indirectly ionized by being absorbed into a stable atom, causing it to become unstable and more likely to emit ionizing radiation of another kind. In fact, neutrons are the only form of radiation capable of making other materials radioactive (Garcia‐Sanchez et al., 2018). Radiation inactivation of viral pathogens using neutrons has potential applications in sterilization and the processing of biological reagents, including noninfectious viral antigens. Experiments were planned to look at direct radiation damage caused by gamma photons (gamma) and neutrons while keeping methodological variations to a minimum. Frozen influenza AX31/H3N2 and PR8/H1N1 samples were subjected to gamma and neutron doses ranging from 0 to 15.6 kGy (Lowy et al., 2001).

### 3.6 Plasma

Plasma is made up of ions, atoms with some of their orbital electrons withdrawn, and free electrons in a gaseous state. Heating or exposing a neutral gas to a strong electromagnetic field to the point that an ionized gaseous material becomes increasingly electrically conductive may be used to create plasma. Long‐range electromagnetic fields affect the resulting charged ions and electrons, making plasma dynamics more sensitive to these fields than a neutral gas would be (Morozov, 2013). Plasma and ionized gases have properties and behaviors that are distinct from those of other states of matter, and the transition between them is largely a matter of terminology and understanding. Plasma exposure of energetic neutrals (such as atoms, molecular components), which includes ions, radicals, photons, and electrons, will modify the surface without changing the bulk properties. Plasma surface‐modification, plasma deposition, and plasma‐induced polymerization or grafting are all examples of plasma processes. Plasma's nondirectional design has an effect on throughput and treatment properties. Surface changes or modifications can be made in a variety of ways. When applied in air, corona treatment (such as corona discharge, and dielectric barrier discharge) involving ozone, electrons, ions, excited molecular species, and radicals causes surface oxidation and significant chain scission, resulting in the formation of water‐soluble low‐molecular‐weight oxidized material (Silvestre et al., 2017). The number of microorganisms (such as bacteria, and fungi) on the surface of foods treated with cold plasma discharges is reduced. Plasma may also be used to decompose a variety of harmful chemical compounds commonly found in food, such as pesticides and mycotoxins (Bartos et al., 2017). Ionizing radiation applications in materials processing involve electrons, photons, atoms, negative ions, fragments, and molecules interacting with a material's surface. The effects of plasma treatment differ depending on the fluence of these moieties and the plasma requirements (such as type of gas and pressure, power, and duration). The higher the free‐radical concentration obtained by uti stronger conditions, for example, the prolonged the plasma treatment. Plasma treatment has a variety of effects on the modified surface, including functionalization, crosslinking, and cleaning depending on the experimental conditions. It has been stated that CO_2_, H_2_O, and CO_2_/H_2_O plasma can be used to modify or functionalize the surface of polyethylene. Except for polyethylene and polypropylene, which have a highly oxidized surface layer, a simple ablation of the surface layer has been observed in several cases. In the emerging field of antimicrobial and bioactive packaging, plasma treatment has been used to immobilize bioactive functional compounds such as lysozyme, glucose oxidase, niacin, vanillin, sodium benzoate, or antimicrobial peptides on a packaging material surface (Silvestre et al., 2017).

Cold‐plasma technology is also a new, environmentally friendly process with a wide range of possible applications in food packaging. Although it was primarily designed to increase the surface energy of polymers in order to improve printability and adhesion, it has recently emerged as a powerful tool for surface disinfection of either foods or food packaging materials. New trends seek to improve in‐package decontamination by providing food post‐packaging nonthermal treatment (Pankaj et al., 2014). Cold plasma processes seem to be well suited for use in the textile industry as well. In reality, they are dry processes with low chemical requirements; in many cases, the desired effects can be achieved with only air, nitrogen, oxygen, or other inert gases. Furthermore, these processes are fast, are flexible, and operate at room temperature, reducing the amount of energy required to heat water or initiate chemical reactions (Lebrun, 2015). According to a recent report, plasma can destroy viruses in seconds, potentially changing the way hospital infections are handled. Plasma could be used to combat diseases like HIV, the common cold, and even cancer. The advantages of cold plasma, such as its environmental friendliness, innovations in textile production, growing food safety issues, developments in cold plasma technology, and the use of cold plasma in meat and poultry packaging decontamination, are mainly responsible for the market's growth (Filipic et al., 2020).

### 3.7 Ozone gas

When high‐energy ultraviolet rays break atmospheric oxygen (O_2_) bonds, forming free‐radical oxygen atoms, which then react with other O_2_ molecules to form ozone (O_3_), ozone is created generally, especially in the upper atmosphere. Natural sources of ozone include lightning storms, waterfalls, and ocean beaches (Cheng et al., 2020). Electrical discharges of high voltage in the air or pure oxygen are the most common sources of ozone formation. To generate ozone, ultraviolet radiation (with a wavelength of 188 nm) and corona discharge methods is used to induce free‐radical oxygen production. The corona discharge method is commonly used to produce commercial quantities of ozone. Since ozone degrades spontaneously to create a free‐radical oxygen atom, it has a high oxidizing capacity. Its antiviral, bactericidal, and fungicidal properties are derived from this oxidizing ability, and as a result, it has a high capacity to disinfect and sterilize (Augusto Goncalves, 2009). Compared to other biocidal agents including chlorine, the time required to disinfect is shorter. In humans, ozone can irritate nasal passages and cause nausea, and long‐term exposure can cause to lung inflammation (Cheng et al., 2020). Ozone is unstable under normal pressure and temperature conditions. Temperature and humidity exacerbate this instability. Ozone technology has many major advantages over chemical alternatives, including the potential to produce ozone on‐site, a high level of operation, and immediate availability. Ozone decomposes quickly to oxygen with no traces left behind, and reactions do not contain toxic halogenated compounds. Ozone disinfects faster and more thoroughly than other traditional disinfectants, and it works on all virus strains quickly and efficiently (Augusto Goncalves, 2009).

The ozone concentration is retained at a fixed amount for a predetermined period of time during sterilization procedure, and, after that time has passed, the ozone is depleted. The ozone generator signals when the ozone concentration falls below a predetermined safe level. Ozone's structure is extremely reactive, and it has a short half‐life as a result (about 30 min). When ozone depletes, oxygen and a free radical oxygen atom are produced. This free radical of oxygen is a potent oxidant. The present invention includes portable equipment, requirements, and operating procedures for providing sufficient ozone exposure in indoor spaces to achieve an efficient degree of sanitization, accompanied by rapid removal of the ozone and associated gaseous byproducts from ozone reactions with carpet and furniture fabrics. Identifying the variables that influence the safe and efficient use of ozone as a disinfectant in the hospitality and other industries is part of the innovation (Cheng et al., 2020). Experts are concerned about toxic by‐products (including dioxins or tri‐halomethanes) formed when chlorine reacts with organic matter in water. Ozone treatment of organic wastes can be considered an efficient and faster process than other treatments with chlorine or hydrogen peroxide. Chlorinated wash systems often necessitate the transportation and storage of potentially dangerous, harmful chemicals. In low concentrations, ozone is not harmful, but at higher concentrations it is an unpleasant gas with poisonous effects on humans (Augusto Goncalves, 2009).

## 4 NONIONIZING RADIATION

In the quest for new and improved food storage and packaging approaches, scientists have looked into the possibility of using nonionizing radiations in various frequency ranges. This type of radiation does not have enough energy to sever molecular bonds or expel electrons from atoms, which is what ionization is all about. Microwave, ultraviolet, infrared, laser, radiofrequency, and visible light are examples of nonionizing wavelengths (Figure 1, Table 3) (Lima et al., 2018).

**TABLE 3:** Applications and observation effects of nonionizing radiations in food processing and packaging

Type	Applications		Observation effects	References
Microwave	Inexpensive tool for sterilization in the pharmaceutical industry, and in drying and thawing in the food industry. Overall temperature attained is effective on viruses, but high enough to denature organic material		Microbial cell death, change in the secondary and tertiary structure of microorganism proteins. Two minutes of microwaving on full power mode killed or inactivated more than 99% of all the living pathogens in tested sponges and pads	Lima et al., 2018; Filipic et al., 2020
Ultraviolet (UV)	UV‐A 400–320 nm	High germicidal capacity, effective disinfection method for microbiological inactivation, medical applications, and treatment plants	Kill or inactivate microorganisms UV destroys viruses when high‐energy electrons pass or diffuse through the protein coat into the nucleic acid core, resulting in damage of the viral RNA Inactivation of H5N1 and coronaviruses, especially by UV‐C or UVGI	Cheng et al., 2020; Lima et al., 2018; Kim and Kang, 2018
	UV‐B 320–280 nm	High germicidal capacity, medical applications		
	UV‐C 280–200 nm	High disinfection and germicidal capacity in food, air, and water purification. UV‐C can be coupled with a filtration system to sanitize air and water		
Infrared (IR)	IR heating has been applied in drying, baking, roasting, blanching, pasteurization, and sterilization of food products		IR inactivation of *Staphylococcus aureus*, a pathogenic microorganism, in drinks such milk was studied to investigate the potential of this technology for milk pasteurization and inactivating foodborne viruses Thermal effect has low inactivation of most surrogates in low‐water‐activity foods, inactivating foodborne viruses including human NoV, HAV, MNV, TuV, FCV, and HEV	Krishnamurthy et al., 2008; Bosch et al., 2018; Bramson, 1968
LASER	Many applications in food processing, packaging, food treatment, and handling environment		Destroys microorganisms, viruses, and bacteria such as HIV. Controls hospital infections such as MRSA and gram‐positive and gram‐negative bacteria in both vegetative form and spores as well as in biofilms, mammalian viruses and bacteriophages, fungi and yeasts, and parasitic protozoa	Shenkenberg, 2007; Batt & Batt, 2014; Kingsley et al., 2018
Radiofrequency (RF)	RF heating provides a rapid heating rate and volumetric heating, resulting in a shorter use time RF has the potential to disinfect and/or disinfest food, agricultural, and environmental materials Rapid pulses of RF are lethal to arthropod pests and may provide a nonthermal disinfestation process for fresh, temperature‐sensitive commodities, as well as a promising alternative to the fumigant methyl bromide		The nonuniform heating of RF as cold‐shock treatment could enhance the bactericidal efficiency and reduce the heating severity necessary for RF processing, thus improving quality retention of low‐moisture foods	Lagunas‐Solar et al., 2006; Zhao et al., 2017; Wei et al., 2018

### 4.1 Microwave radiation

Microwave energy (300 MHz‐300 GHz) has been thoroughly researched as a potential alternative energy source for sterilization. Microwave radiation is insufficiently energetic to ionize food molecules or produce free radicals. The basic concept of the method is molecular vibration baking, which penetrates the foods superficially with a depth of penetration ranging from 2 to 4 cm. The process vibrates the food, heating the water, fat, and sugar molecules as a result. Microwave heating, on the other hand, causes temperature distribution in the product to be irregular. This heat also clearly causes what is called denaturation of organic molecules, rendering them dysfunctional. This would be an ideal situation, but, in practice, viruses are most often mechanically occluded, either trapped inside another material, or even just located on a certain surface that absorbs heat away from the target. Microwave sterilization ultimately depends on overall temperature attained, which must be high enough to denature organic material. Finally, microwave‐sterilized products do not exhibit nonenzymatic darkening or the formation of a surface crust, which may be beneficial in certain applications. Microwave irradiation was used in experiments, and it was discovered that microbial cell death occurs as a result of the heat generated by the irradiation as well as the electric field formed by the microwaves, which promotes the secondary and tertiary structure changes in microorganism proteins. Microwave energy is currently used in the pharmaceutical industry, as well as in the food industry for drying, thawing, and sterilization processes (Filipic et al., 2020). Despite the shortcomings of this technology, the use of microwave energy has benefits in terms of utensil decontamination and process time, resulting in lower energy costs and the elimination of chemicals typically used in some traditional processes. Both of these factors make it economically and environmentally beneficial to apply this technology in industrial systems, given that its disadvantages are considered, such as the lack of uniformity in heating delivery in systems lacking temperature and power control (Lima et al., 2018).

### 4.2 Ultraviolet radiation

UV light (ultraviolet light) is the term we use to describe light with wavelengths lower than visible light. Violet is the color of the highest frequency of visible light. The wavelengths of visible light are generally described as being between 400 and 700 nanometers. UV light is commonly defined as having a wavelength range of 100–380 nm in a three‐wavelength area (Cheng et al., 2020). UV light has a broad wavelength range in the nonionizing region of the electromagnetic spectrum, occupying a region between X‐rays (100 nm) and visible light (400 nm). UV light is divided into three wavelength bands: UV‐A, UV‐B, and UV‐C. In sunlight, the long (UV‐A, 400–320 nm) and middle (UV‐B, 280–320 nm) wavelengths have germicidal properties. Short wavelengths, or UV‐C (200–280 nm), have a high germicidal potential, but they do not occur naturally and must be created by the conversion of electric energy. UV‐C has the potential to be proven better than other preservation techniques at an acceptable dose, which can help in maintaining the desired quality, enhancing the nutritive value of the product during storage, and has the efficacy of eliminating COVID‐19, according to data collected from existing studies (Bisht et al., 2021). At wavelengths approaching 253.7 nm, UV is especially susceptible to the virus (UV‐C range). Because a DNA molecule's maximal absorption wavelength is about 260 nm, this is the case. To be effective against viruses, UV‐A and UV‐B require greater exposure duration (Quevedo et al., 2020).

UV light can be used in either a continuous or pulsed mode; the pulsed mode is more efficient for microbiological inactivation and is the most commonly used process (Lima et al., 2018). It is important to remember that any commercial UV light source produces a spectrum of UV light, not just one wavelength. UV light with a wavelength of 185 nm is “tuned” to produce UV light at 185 nm, but it can also produce UV light with a wavelength of 100–240 nm. Ultraviolet germicidal irradiation (**UVGI**) is a disinfection process that uses short‐wavelength ultraviolet (UV‐C) light to kill or inactivate microorganisms by destroying nucleic acids and disrupting DNA, rendering them unable to perform vital cellular functions. UVGI is used in a number of applications, including the purification of food, air, and water. Since the ozone layer of the atmosphere absorbs UV‐C radiation, it is also thin at the Earth's surface. UVGI devices can generate enough UV‐C light in circulating air or water systems to make them inhospitable to bacteria, viruses, molds, and other pathogens. To sanitize air and water, UVGI can be combined with a filtration device (Cheng et al., 2020).

Viruses are tiny, self‐contained particles made up of crystals and macromolecules that replicate only inside the host cell, unlike bacteria. They convert proteins from the host cell into proteins of their own. UV kills viruses by passing or diffusing high‐energy electrons through the protein coat into the nucleic acid core, damaging the viral RNA. The inactivation rate constants of each microorganism were determined in fitting curves of surviving populations using UV‐C light‐emitting‐diode (LED) arrays. For MS2, Qβ, and ϕX174 viruses, UV‐C LED array treatment effectively inactivated viral infectivity. The UV‐C LED array was also effective in inactivating *Salmonella enterica* serotype typhimurium and *E. coli* O157:H7 (Kim and Kang, 2018).

### 4.3 Ultraviolet disinfection

Proteins, RNA, and DNA in a given microorganism absorb ultraviolet light. UV absorption by proteins in membranes at high fluency (UV dose) eventually resulting in cell membrane disruption and, as a consequence, cell death. However, at much lower fluencies (UV doses), UV absorption by DNA (or RNA in some viruses) can disrupt the microorganism's ability to replicate. A cell that is unable to replicate is incapable of causing disease. Some microorganisms (particularly bacteria) have a thymine dimer dissociation repair mechanism. The absorption of UV light initiates this process, which is referred to as photo reactivation. The repair mechanism can be bypassed, but it will take more fluency. UVGI is successful in inactivating H5N1 influenza and coronaviruses, according to the above information (Cheng et al., 2020). Under laboratory‐controlled conditions, determining the sensitivities of all hazard viruses to UV_254_ in the UV‐C range and to radiation from UV‐A/B solar simulators revealed that UV_254_ inactivated selected viruses as virus particles suspended in an aqueous solution, on surfaces, and aerosolized (Grant, 1997). UV light is a physical process that has many benefits, including the absence of by‐products that may alter food quality, the absence of chemical traces or ionizing radiation, and the fact that it is a cold process, dry, simple, efficient, and low‐cost in comparison to other sterilization methods (Lima et al., 2018). UV light has thus been used in water purification and beverages industries; in the food industry, it has been used in final washing, CIP (Clean in Place) machine rinsing water, and the disinfection of storage tanks and packaging surfaces.

#### 4.3.1 Ultraviolet and ozone production

**FIGURE 3:**
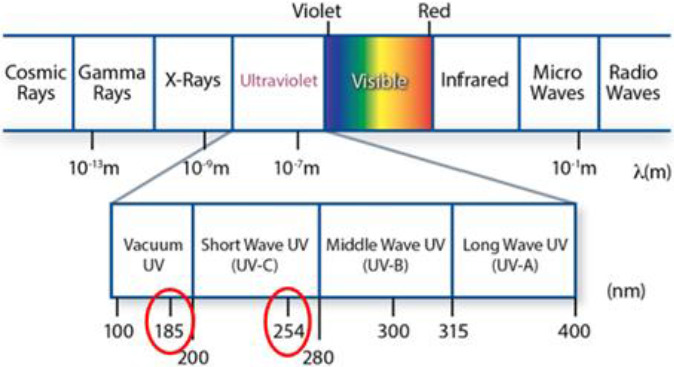
The category of UV spectrum by emphasize on germicidal efficiency without ozone production (Cheng et al., 2020)

For a heating, ventilation, air conditioning, and refrigeration (**HVACR**) unit, the ultraviolet germicidal irradiation (i.e., UV‐C) wavelength is an indispensable method. Facility managers profit from reducing the dissemination of airborne infectious agents by using germicidal energy to maintain refrigeration coils clean of microbial development. However, some facility administrators may be unable to take advantage of these opportunities because of ozone concerns. Though there are four distinct wavelengths in the ultraviolet spectrum (UV‐A, B, C, and vacuum‐UV), each acts at a particular energy level, and only one is capable of creating ozone (vacuum‐UV). UV‐C, on the other hand, achieves its maximum germicidal intensity around 253.7 nm, while vacuum‐UV is capable of creating ozone and works in the 100–200 nm range. Since ozone can only be generated at wavelengths below 200 nm, the germicidal wavelength of 253.7 nm (rounded to 254 nm) does not produce ozone, as seen in Figure 3. UV‐C lamps have an additional layer of ozone defense in comparison to the stronger 254‐nm wavelength that does not emit ozone (Cheng et al., 2020).

### 4.4 Infrared radiation

The infrared spectrum spans about 300 GHz to 400 THz (750 nm–1 mm). It is divided into three main parts (Bramson, 1968):

- **Far‐infrared**: Far‐infrared wavelengths ranges from 300 GHz to 30 THz (1 mm to 10 µm). Microwaves and terahertz waves are terms used to describe the lower end of this spectrum. Rotational modes in gas‐phase molecules, molecular motions in liquids, and phonons in solids are the most common absorbers of this radiation.
- **Mid‐infrared**: Mid‐infrared wavelengths ranges from 30 to 120 THz (2.5–10 µm). Hot artifacts (black‐body radiators) can radiate intensely in this range, and human skin radiates strongly at the lower end of this range at average body temperature. This energy is consumed by molecular vibrations, which occur as the atoms in a molecule vibrate in their equilibrium positions.
- **Near‐infrared**: Near‐infrared, ranging from 120 to 400 THz (750–2500 nm). The physical mechanisms that apply to this spectrum are identical to those that apply to visible light. Some varieties of photographic film and several types of solid‐state image sensors for infrared imaging and videography can detect the highest frequencies in this area directly.

Infrared (IR) heating has many benefits over traditional heating, including shortened heating times, more uniform heating, lower quality losses, no solute migration of food content, flexibility, simplicity, lightweight and compact facilities, and substantial energy savings. Drying, frying, roasting, blanching, pasteurization, and sterilization are only a few of the processes that may benefit from infrared heating. Because of improved energy throughput, combinations of infrared heating with microwave heating and other traditional conductive and convective heating modes have been gaining traction (Krishnamurthy et al., 2008). The effectiveness of infrared heating for inactivating *Staphylococcus aureus*, a pathogenic microorganism, in liquids like milk, for example, was investigated to see whether this technology could be used for milk pasteurization. The findings showed that infrared heating has a high propensity for successful S. aureus inactivation in milk. Further process optimization could lead to an economically feasible milk pasteurization method (Bramson, 1968). IR radiation has now been commonly used in the food industry for a variety of thermal processing activities, including dehydration, baking, frying, and pasteurization.

### 4.5 IR and thermal processing

Thermal processing has shown to be an important method of inactivating foodborne viruses such as human NoV, HAV, and HEV. Heating or treating with IR radiation wavelengths may be used for thermal processing. Also, in complex matrices like shellfish, temperatures above 90℃ are normally successful against enteric viruses. Furthermore, after 15 min at 60℃, human NoV GII.3 and GII.4 stool suspensions lost infectivity to stem cell–derived human enteroids, demonstrating the efficacy of heat as an inactivation technique for enteric viruses. Hirneisen and Kniel (2013) found that heating MNV and TuV to 70℃ for 2 min inactivated them above the detection point, and that NoV surrogates acted similarly during heat treatment. Blanching spinach at 80℃ for 1 min, a popular industrial procedure, decreased infectious MNV by at least 2.4 log10. Both HAV and FCV were inactivated by steam blanching various herbs at 95℃ for 2.5 min. Deboosere et al. (2010) established a thermal inactivation model for HAV in red berries at various pH values and found that in the measured pH range of 2.5–3.3, lower pH resulted in quicker inactivation. Using a cell culture–based procedure, Barnaud et al. (2012) demonstrated that heating pork meat to an internal temperature of 71℃ for 20 min was needed to inactivate HEV, and heating at 70℃ for 2 min in buffer resulted in no observable virus (over 3.9 log10 decrease).

### 4.6 Laser

Light amplification by the stimulated emission of radiation (**Laser**) is a mechanism that causes atoms or molecules to emit light at certain wavelengths and amplifies that light, creating a very narrow beam of radiation. The emission typically only contains a small spectrum of visible, infrared, and ultraviolet wavelengths. There have been several different types of lasers produced, each with its own set of characteristics. In this article, we briefly discuss some of the technology's uses in the areas of bacteria and virus care, food production, and packaging. A new laser approach could make it possible to treat drug‐resistant viruses and bacteria, such as HIV and methicillin‐resistant *Staphylococcus aureus* (**MRSA**), in a safe and efficient manner. Existing laser therapies, such as ultraviolet irradiation, avoid the issue of drug resistance, but they are also associated with health side effects. For example, ultraviolet radiation is known to cause cancer and, if not used properly, will kill healthy cells while killing pathogenic microorganisms. Instead of using these methods, the latest approach employs impulsive induced Raman scattering, which uses vibrations from a femtosecond laser to split the bonds between proteins in microorganism protein coats. As a result, the femtosecond laser destroys the protein coat in a manner similar to glass shattering. This approach prevents dangerous ultraviolet radiation since femtosecond lasers work at safe, near‐infrared wavelengths. Physicists at Arizona State University have now developed a novel laser tool that can kill viruses and bacteria such as AIDS without harming human cells; this may help minimize the spread of hospital infections such as MRSA. Current laser treatments, such as UV, are indiscriminate, can induce skin aging, DNA destruction, or, in the worst‐case scenario, skin cancer, and are far from entirely successful. The physicists in Arizona conducted studies to demonstrate that coherent vibrations excited by infrared lasers of precisely chosen wavelengths and pulse widths do little harm to human cells, most likely due to differences in the structural compositions of human cell protein coats compared to bacteria and viruses. Although it is unclear why there is a significant gap in laser strength for inactivation of human cells versus microorganisms such as bacteria and viruses, current research indicates that impulsive stimulated Raman scattering (**ISRS**) would be ready for use in disinfection and may offer therapies against some of the most dangerous, often drug‐resistant, bacterial and viral pathogens (Shenkenberg, 2007). Femtosecond lasers could be used immediately in hospitals to clean blood supplies and biomaterials, as well as to treat blood‐borne diseases like hepatitis and AIDS (Institute of Physics, 2007). The findings indicate that visible laser light causes the production of short‐lived reactive oxygen species including singlet oxygen, rather than an ISRS process that produces resonant vibrations, as the cause of virus inactivation (Kingsley et al., 2018). A diverse variety of microorganisms (such as gram‐positive and gram‐negative bacteria including both vegetative and spore form, and also in biofilms, fungi, and yeasts, parasitic protozoa, and mammalian viruses or bacteriophages) were shown to be susceptible to photosensitization‐based treatments. Laser technology, on the other hand, has many uses in pharmacy, food manufacturing, and other industries; it is a more efficient and stable approach than other technologies (Luksiene & Brovko, 2013).

### 4.7 Radiofrequency

Radiofrequency (RF) is a high‐tech telecommunications system that was first developed in the early 1900s and is still used for cellular networking around the world today. RF has the capacity to disinfect and/or disinfest foodstaffs, agricultural, and environmental materials due to its ability to penetrate and heat different materials. Even so, validation research has been hampered by a lack of knowledge of how RF photons interact with substances, as well as inadequate access to and high costs of source electronics. Fresh produce, grain, soils, farm wastewater, and other foods and products have all been successfully disinfected and/or disinfested using RF power in laboratory experiments. Rapid RF pulses are often lethal to arthropod pests, suggesting that they may be used as a nonthermal disinfestation method for new, temperature‐sensitive products, as well as a potential alternative to the methyl bromide as the common fumigant. RF heating is a widely used food processing technology that has been used for food drying, frying, baking, thawing, sterilization, and disinfection. Electromagnetic waves are used in RF processing to thermally treat foods using dielectric heating (Lagunas‐Solar et al., 2006). After studying the microbial reduction and color alteration in broccoli powder after RF heating for various time periods, it was discovered that using cold shock in combination with RF heating is a promising technology that has the ability to minimize the strength of applied RF, resulting in improved quality preservation of low‐moisture foods (Zhao et al., 2017).

## 5 CONCLUSION

- Different radiation and optical technologies, including UV, laser, and high‐intensity pulsed light (PL), as well as microwaves, have been investigated for use in disinfection and treatment in the food processing industry. The effective implementation of these technologies is dependent on light immediately hitting all virus particles; if viruses are found in holes, crevices, or gaps in the foodstaffs or materials surfaces, these viruses may be protected from light exposure and hence survive light‐treatment.
- The ultraviolet ranges are used in the majority of photochemistry experiments. The exposure of human skin to ultraviolet light is divided into three sub‐ranges. The UV‐A spectrum induces skin changes that result in sun tanning. UV‐B rays can cause sunburn and are linked to the development of skin cancer. UV‐C radiation is highly toxic because it is absorbed by proteins, RNA, and DNA, causing cell mutation, cancer disease, and eventually death. Because it is so effective at inactivating bacteria and viruses, the UV‐C range is often referred to as the germicidal range. The vacuum ultraviolet (VUV) spectrum is a third range that nearly all compounds absorb (such as water and air). As a result, it can only be sent in a vacuum. A VUV photon's absorption induces one or two bond breaks.
- Since the word "dose" is used to mean total absorbed energy ( for example, the "UV dosage" needed to produce sunburn on the skin), the term "fluency" is favoured over "UV dose." Fluency is the radiant energy “incident” on a microorganism, although only a small fraction (approximately 1% ) of the energy is absorbed in most situations.
- The new electromagnetic disinfection‐sterilization technology involves repeatedly exposing the product to high‐intensity fields with short electrical pulses (ms or μs) in order to inactivate enzymes and kill microorganisms and viruses. Corona discharge for ozone producing and plasma are two similar processes used in the food industry.

## CONFLICT OF INTEREST

The authors declare that they have no conflict of interest and that no human or animal research was included in the report.

## AUTHOR CONTRIBUTION

**Sharifeh Shahi:** Conceptualization (lead); Data curation (lead); Formal analysis (lead); Funding acquisition (lead); Investigation (lead); Methodology (lead); Project administration (lead); Resources (lead); Software (lead); Supervision (lead); Validation (lead); Visualization (lead); Writing‐original draft (lead); Writing‐review & editing (lead). **Reza Khorvash:** Conceptualization (equal); Data curation (equal); Formal analysis (equal); Funding acquisition (equal); Investigation (equal); Methodology (equal); Project administration (equal); Resources (equal); Software (equal); Supervision (equal); Validation (equal); Visualization (equal); Writing‐original draft (equal); Writing‐review & editing (equal). **Mohammad Goli:** Conceptualization (lead); Data curation (lead); Formal analysis (lead); Funding acquisition (lead); Investigation (lead); Methodology (lead); Project administration (lead); Resources (lead); Software (lead); Supervision (lead); Validation (lead); Visualization (lead); Writing‐original draft (lead); Writing‐review & editing (lead). **Mohsen Ranjbaran:** Conceptualization (equal); Data curation (equal); Formal analysis (equal); Funding acquisition (equal); Investigation (equal); Methodology (equal); Project administration (equal); Resources (equal); Software (equal); Supervision (equal); Validation (equal); Visualization (equal); Writing‐original draft (equal); Writing‐review & editing (equal). **Afsaneh Najarian:** Conceptualization (equal); Data curation (equal); Formal analysis (equal); Funding acquisition (equal); Investigation (equal); Methodology (equal); Project administration (equal); Resources (equal); Software (equal); Supervision (equal); Validation (equal); Visualization (equal); Writing‐original draft (equal); Writing‐review & editing (equal). **Abdorreza Mohammadi Nafchi:** Conceptualization (equal); Data curation (equal); Formal analysis (equal); Funding acquisition (equal); Investigation (equal); Methodology (equal); Project administration (equal); Resources (equal); Software (equal); Supervision (equal); Validation (equal); Visualization (equal); Writing‐original draft (equal); Writing‐review & editing (equal).

## DATA AVAILABILITY STATEMENT

The data that support the findings of this study are available from the corresponding author upon reasonable request.
